# Thiamine Mitigates Nicotine Withdrawal Effects in Adolescent Male Rats: Modulation of Serotonin Metabolism, BDNF, Oxidative Stress, and Neuroinflammation

**DOI:** 10.1523/ENEURO.0140-25.2025

**Published:** 2025-08-14

**Authors:** Murtaza Haidary, Elham Akbari, Mohammad Edris Amiri, Khan Baba Ghazanfar, Mohammad Tariq Anwary, Mohammad Jalal Nazari, Mohammad Hussain Khadimi

**Affiliations:** ^1^Medical Research and Technology Center, Khatam Al-Nabieen University, Kabul 1001, Afghanistan; ^2^Department of Biology and Microbiology, Faculty of Medical Laboratory Technology, Khatam Al- Nabieen University, Kabul 1001, Afghanistan; ^3^Department of Internal Medicine, Faculty of Medicine, Khatam Al-Nabieen University, Kabul 1001, Afghanistan

**Keywords:** adolescence, anxiety, depression, neuroinflammation, nicotine withdrawal, oxidative stress

## Abstract

Adolescent nicotine use is particularly concerning due to increased susceptibility to long-term effects and dependence during this critical developmental period. This study investigates the therapeutic effects of thiamine on nicotine withdrawal-induced anxiety, anhedonia, and depression in rats. Adolescent rats received nicotine (2 mg/kg, s.c.) for 21 d, followed by 21 d of withdrawal. Thiamine (25 or 50 mg/kg, i.p.) was administered during exposure and withdrawal. Behavioral assessments were used to evaluate anxiety- and depressive-like symptoms, and biochemical analyses measured oxidative stress markers, serotonin levels, MAO activity, BDNF, and GFAP as indicators of neuroinflammation in the prefrontal cortex. Nicotine withdrawal significantly elevated anxiety-, depression-, and anhedonia-like behaviors, increased oxidative stress, and upregulated MAO-A activity and GFAP expression, indicating neuroinflammatory effects. Notably, thiamine administration during both nicotine exposure and withdrawal effectively alleviated these behavioral impairments, restored serotonin levels, reduced oxidative stress markers, and mitigated the increase in GFAP expression. Additionally, thiamine alone has been shown to alleviate anxiety- and depressive-like behaviors. This study highlights thiamine's potential as a promising intervention for managing psychological distress associated with nicotine withdrawal. Given the high prevalence of adolescent nicotine use and its associated mental health challenges, further research on thiamine's mechanisms and therapeutic potential is warranted to improve treatment strategies during this critical developmental stage.

## Significance Statement

Nicotine dependence remains a global health challenge, with withdrawal symptoms posing a significant barrier to cessation efforts, particularly in adolescents. This study explores the neuroprotective potential of thiamine in mitigating nicotine withdrawal in adolescent male rats by modulating key neurochemical pathways. Thiamine treatment was found to attenuate withdrawal-associated behavioral disturbances, while also restoring serotonin metabolism, upregulating brain-derived neurotrophic factor, and reducing oxidative stress and neuroinflammation. These findings highlight thiamine's multifaceted role in alleviating nicotine withdrawal by addressing both neurochemical and inflammatory imbalances. This study suggests that thiamine could serve as an adjunct therapeutic approach for nicotine dependence, with implications for improving cessation outcomes during adolescence—a critical period for brain development and vulnerability to dependence.

## Introduction

Adolescent nicotine use remains a significant public health concern due to the heightened risk of addiction and increased vulnerability to the long-term adverse effects of nicotine exposure during this developmental stage (Hossaini et al., 2025b). During this critical stage of development, the central nervous system undergoes significant structural and functional changes (Baum et al., 2022), alongside major modifications in neurotransmitter systems associated with reward processing (Bonke et al., 2023). These neurological changes contribute to adolescent-specific behaviors, including increased novelty-seeking and risk-taking (Kim-Spoon et al., 2019). Nicotine, a psychostimulant with parasympathomimetic effects, shares pharmacological properties with amphetamines, increasing its potential for abuse and addiction (Motaghinejad et al., 2016). Chronic nicotine exposure enhances reward processing mechanisms, contributing to its high addiction potential across various tobacco products (Torabi et al., 2019). Adolescents have a lower sensitivity to nicotine's aversive effects, potentially contributing to its higher use in this population (von Deneen et al., 2022). There is a well-documented link between nicotine addiction and psychiatric disorders, particularly anxiety (Feldner et al., 2007; Jobson et al., 2019) and depression (John et al., 2004; Amiry et al., 2023). Anxiety disorders are significantly more prevalent among individuals with nicotine dependence, affecting ∼22% of this population compared with only 11.1% of nondependent individuals (Grant et al., 2004). Nicotine metabolism generates reactive oxygen species (Jobson et al., 2019), leading to oxidative stress that induces cellular damage and contribute to behavioral disorders, including anxiety, depression, neuroinflammation, and neurotransmitter imbalances (Paz et al., 2007; Ahmadi-Soleimani et al., 2023; Amiry et al., 2023).

While pharmacological treatments for nicotine withdrawal exist, research increasingly seeks safer and more effective alternatives (Yammine et al., 2021). Nutritional psychiatry has gained attention for its investigation into how dietary modifications and specific micronutrient supplementation influence mental health, particularly mood disorders (Botturi et al., 2020). Vitamins, as essential organic compounds, act as cofactors in enzymatic reactions; since mammalian cells are typically unable to synthesize them, they must be obtained through dietary intake (Fattal-Valevski, 2011). Thiamine (vitamin B1) is a water-soluble vitamin crucial for neuronal function and energy metabolism (Butterworth, 2003). Its depletion, which can occur within weeks of inadequate intake, has been linked to behavioral impairments in young individuals (Butterworth, 2003). This study aims to evaluate the effects of thiamine administration on anxiety- and depression-like behaviors resulting from nicotine dependence, particularly during the withdrawal period following nicotine exposure. Additionally, it investigates the impact of concurrent nicotine and thiamine administration on the development of these behavioral alterations in rats.

## Materials and Methods

### Animals

Eighty adolescent male Sprague Dawley rats were selected for this study to reduce biological variability and eliminate confounding hormonal influences related to the estrous cycle, which can affect behavioral and neurochemical responses during nicotine withdrawal (Becker et al., 2005; Carroll and Anker, 2010). Female rodents often exhibit milder withdrawal symptoms—particularly in measures of anxiety, hyperalgesia, and locomotor activity—which may obscure the detection of treatment effects (Chellian et al., 2021). Male adolescents are widely used in nicotine dependence research due to their consistent and well-characterized withdrawal profiles (O'Dell et al., 2007), providing greater experimental control in mechanistic investigations. Rats were obtained from the animal facility of Khatam Al-Nabieen University at postnatal day 21 (P21) and housed in groups of three to four per open-top plexiglass cage. The rats were kept under standardized environmental conditions, featuring a controlled temperature of 22 ± 2°C, humidity levels maintained between 55 and 65%, and a 12 h light/dark cycle, with lights turning on at 6:00 A.M. The animals received a standard laboratory diet (Javaneh Khorasan) containing 46% nanofibrillated cellulose, 25% neutral detergent fibers, 19% protein, and 10% lipids, with *ad libitum* access to clean water. All experimental procedures were approved by the Animal Ethics Committee of the author's university (AF, knu.edu.af.rec 23, 10/5/2024) and were conducted in strict accordance with the Guide for the Care and Use of Laboratory Animals (Ahmadi-Noorbakhsh et al., 2021).

### Drugs and experimental procedure

**Figure 1.**
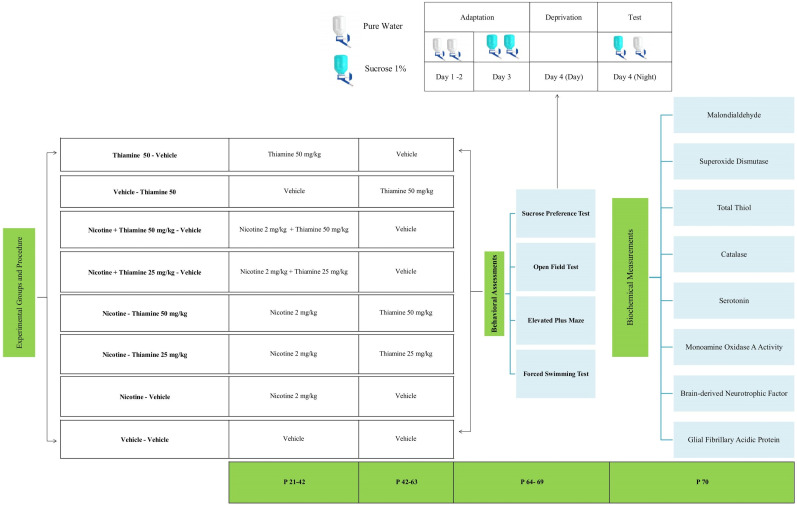
A detailed timeline outlining the key phases and experimental events in the study. P, postnatal day; OFT, open field test; EPM, elevated plus maze; FST, forced swimming test; SPT, sucrose preference test; MDA, malondialdehyde; TT, total thiol; SOD, superoxide dismutase; CAT, catalase; MAO-A, monoamine oxidase A; BDNF, brain-derived neurotrophic factor; GFAP, glial fibrillary acidic protein.

(−)Nicotine base and thiamine were obtained from Sigma-Aldrich and diluted in normal saline (0.9% sodium chloride) for administration and were injected into a final volume of 1 ml. Rats received subcutaneous (s.c.) nicotine injections at a dose of 2 mg/kg every 12 h (6:00 A.M. and 6:00 P.M.). This model is widely utilized in preclinical studies to simulate nicotine dependence, as it allows for the controlled evaluation of key behavioral adaptations such as tolerance, reinforcement, and withdrawal symptoms—core features that underlie nicotine addiction and are essential targets for the development of therapeutic interventions (Chellian et al., 2021). The study consisted of eight experimental groups (*n* = 10 per group), detailed as follows: The experimental design consisted of eight groups of adolescent rats (*n* = 10 per group), each receiving specific treatment regimens across two distinct phases—P21–42 (nicotine exposure period) and P42–63 (postexposure period). Group 1 (Vehicle–Vehicle): Served as the control group and received saline injections throughout the entire experimental period (P21–63). Group 2 (Nicotine–Vehicle): Received nicotine (2 mg/kg, s.c.) twice daily during the nicotine exposure phase (P21–42), followed by saline during the postexposure period (P42–63). This group was used to assess the behavioral effects of nicotine administration and spontaneous withdrawal. Groups 3 and 4 (Nicotine–Thiamine): Received nicotine (2 mg/kg, s.c.) during the exposure phase (P21–42), followed by thiamine administration at either 25 mg/kg (Group 3) and 50 mg/kg (i.p.; Group 4), during the postexposure phase (P42–63), to evaluate the potential of thiamine as a postwithdrawal intervention. Groups 5 and 6 (Nicotine + Thiamine–Vehicle): Administered both nicotine (2 mg/kg, s.c.) and thiamine (25 mg/kg for Group 5 and 50 mg/kg for Group 6, i.p.) simultaneously during P21–42, followed by saline injections from P42–63. These groups were included to assess the potential protective effects of thiamine when coadministered with nicotine during exposure. Group 7 (Thiamine–Vehicle): Received thiamine alone (50 mg/kg, i.p.) from P21 to P42, followed by saline from P42 to P63, to evaluate the independent effects of thiamine. Group 8 (Vehicle–Thiamine): Administered saline during the exposure phase (P21–42), followed by thiamine (50 mg/kg, i.p.) during the postexposure phase (P42–63), to determine the efficacy of delayed thiamine treatment. The selected thiamine dosages were informed by previous research demonstrating its neurophysiological benefits (Kim et al., 1990; Karabulut et al., 2022). Furthermore, clinical data indicate that doses ranging from 25 to 50 mg/kg are safely used in human infants and children for the treatment of thiamine deficiency (Smith et al., 2021). The schematic schedule of the experiments is presented in Figure 1.

### Behavioral assessments

During the initial week following nicotine cessation, rats typically do not exhibit significant changes in anxiety-like behavior (Chellian et al., 2021). Therefore, in this study, to evaluate the behavioral consequences of nicotine withdrawal in adolescent rats, a battery of validated behavioral tests—including the open field test (OFT), elevated plus maze (EPM), forced swimming test (FST; Popovych et al., 2019), and sucrose preference test (SPT)—was performed on Day 21 following the final nicotine injection. This time point was selected to capture the intermediate withdrawal phase when emotional and cognitive disturbances are typically evident. Before each behavioral test, rats were allowed to acclimate to the testing environment for at least 30 min to reduce environmental stress and increase data reliability. Illumination levels were standardized according to task requirements: 150 lux for the EPM to encourage open-arm exploration, and 40 lux for the OFT to support natural exploratory behavior without inducing anxiety. To avoid potential confounding effects, animals that had completed behavioral testing were housed separately from those awaiting evaluation. Furthermore, all testing apparatuses were sanitized between trials using a 10% ethanol solution to eliminate olfactory cues from previous subjects, ensuring the consistency and validity of behavioral measurements.

#### Open field test

The OFT is a widely used behavioral assay for assessing anxiety-like behaviors and exploring the neurobiological mechanisms underlying anxiety. It is also employed to evaluate the efficacy of potential anxiolytic compounds (Kraeuter et al., 2019). In this test, an animal is placed in a large, open arena, and its movements are monitored. The test is based on the conflict between the animal's innate fear of open spaces and its curiosity to explore. Time spent in the center of the arena is often interpreted as an indicator of reduced anxiety. More time spent in the center typically suggests lower anxiety levels (Sturman et al., 2018). In this study, the OFT was employed to evaluate anxiety-like behavior. The test was conducted in a square arena measuring 100 × 100 × 40 cm, constructed from opaque, nonreflective material to minimize external visual distractions. Each rat was gently placed in the center of the arena and allowed to explore freely for 5 min. A 5 min duration allows for the collection of reliable data while minimizing the potential for habituation effects, where animals may become accustomed to the environment and alter their behavior over time (Delprato et al., 2017). This time is also long enough to capture the initial anxiety response to a novel environment, which is critical for evaluating the effects of pharmacological interventions or genetic modifications on anxiety and exploratory behavior (Mesiakaris et al., 2024). During the session, behavioral parameters—including the time spent in the central versus peripheral zones—were recorded using an automated video tracking system. The data were subsequently analyzed to assess levels of and anxiety-like behavior.

#### Elevated plus maze

Following the OFT, the anxiolytic effects of thiamine were assessed using the EPM, a well-established method for evaluating anxiety-like behavior and locomotor activity. The EPM consists of a plus-shaped platform elevated 50 cm above the ground, featuring two open arms and two enclosed arms with 40 cm high walls to provide security. Each rat was individually placed in the central area of the maze under dim lighting, allowing free access to any of the arms. The animal's behavior was recorded over 5 min, with particular emphasis on the time spent in each arm. This test leverages the natural conflict in rats between their instinctual aversion to open spaces and their exploratory drive. Anxiety levels were inferred by comparing the time spent in the open versus the closed arms, as rats with lower anxiety levels are more likely to explore the open arms, whereas those with higher anxiety levels tend to remain predominantly in the closed arms (Acikgoz et al., 2022). In this study, the EPM apparatus was constructed from opaque gray wood to minimize external visual cues and enhance the animals’ sense of security during testing. The maze consisted of two open arms and two closed arms arranged in a plus-shaped configuration and elevated above the floor to provoke an approach-avoidance conflict. Each rat was placed at the center of the maze facing one of the open arms and was allowed to explore freely for 5 min. Behavioral parameters, including the time spent in the open and closed arms, were carefully recorded and analyzed. These measurements provided valuable insights into anxiety-like behaviors and were used to evaluate the potential anxiolytic effects of thiamine administration.

#### Forced swimming test

In the FST, rats are placed in a water-filled container from which they cannot escape, and their behavior is monitored to assess depressive-like states. The test records the time spent in various behavioral states, including active behaviors such as swimming and struggling, as well as immobility, which is interpreted as a marker of behavioral despair, analogous to depressive symptoms in humans (Pumpaisalchai et al., 2005). In this study, the FST was employed to assess depression-like behavior in rats. Each animal was individually placed in a transparent glass cylinder (height, 50 cm; diameter, 20 cm) filled with water at a depth of 30 cm and maintained at a temperature of 24 ± 2°C, ensuring that the rats were unable to touch the bottom and were required to swim. The test duration was 5 min, during which each rat was gently introduced into the water and its behavior was continuously recorded using a video camera for subsequent analysis. Behavioral responses were categorized into three distinct types: struggling (vigorous activity involving forelimb and hindlimb movements, including attempts to escape by climbing the walls), swimming (active horizontal movement throughout the container without climbing attempts), and immobility (minimal movement, with only slight motions necessary to keep the head above water). These behavioral parameters were later quantified to evaluate the antidepressant-like effects of thiamine.

#### Sucrose preference test

The SPT is a widely used behavioral assay to assess anhedonia, a core symptom of depression characterized by the inability to experience pleasure. This test evaluates rats’ relative preference for a 1–2% sucrose solution compared with water as an indicator of reward sensitivity (Liu et al., 2018). Rats typically exhibit a preference for sweet, palatable solutions, as sucrose consumption is associated with the activation of reward pathways. A decreased preference for sucrose is therefore considered a sign of anhedonia (Scheggi et al., 2018). This phenomenon is frequently observed in individuals with substance use disorders, major depressive disorder, and various neuropsychiatric conditions, making the SPT a valuable tool for studying the neurobiological mechanisms underlying these disorders in rodents models (Pizzagalli, 2014). In this study, the SPT was conducted to evaluate anhedonia-like behavior, a core symptom of depression. All rats underwent a 4 d adaptation protocol prior to testing. During the first 2 d (Days 1 and 2), animals were provided with two identical bottles containing pure water to minimize any innate side preference. On Day 3, a habituation phase was implemented in which both bottles were filled with a 1% sucrose solution, allowing the rats to become familiar with the sweet taste. On Day 4, following a 12 h period of food and water deprivation, each rat was presented with two bottles: one containing 1% sucrose solution and the other containing pure water. After 12 h of access, the volumes consumed from each bottle were measured to determine sucrose preference. The sucrose preference index was calculated as the ratio of sucrose solution intake to the total fluid intake, providing a quantitative measure of reward sensitivity. Sucrose preference was calculated using the following formula: sucrose preference percentage (%) = sucrose solution consumption / (sucrose solution consumption + water consumption) × 100% (He et al., 2020).

### Euthanasia

Following the behavioral experiments, the rats were killed using a 95% CO_2_ method, employing a step-fill technique in which CO_2_ was introduced into the euthanasia chamber at a rate of 30% of the chamber volume per minute (Begni et al., 2016). After killing, the brains were carefully extracted, and the prefrontal cortex (PFC) was dissected from the surrounding cortical tissue and promptly frozen for subsequent biochemical analyses. These analyses included measurements of serotonin levels, monoamine oxidase A (MAO-A) activity, brain-derived neurotrophic factor (BDNF), glial fibrillary acidic protein (GFAP), malondialdehyde (MDA), total thiol (TT) content, superoxide dismutase (SOD) activity, and catalase activity.

### Biochemical measurements

#### Measurement of serotonin, MAO-A activity, BDNF, and GFAP

Serotonin concentrations were measured using an ELISA kit from My BioSource (MBS713292). MAO-A activity was determined with a separate ELISA kit from the same supplier (MBS721413). BDNF levels were quantified using a rat-specific ELISA kit from CUSABIO (CSB-E04504r). Similarly, GFAP concentrations were assessed with a CUSABIO ELISA kit designed for rat samples (CSB-E08602r). All experimental procedures were conducted in accordance with the manufacturer's guidelines to ensure the accuracy and reliability of the results. Quantitative analysis involved measuring absorbance with a BioTek microplate reader, and the results were compared against a standard curve established under consistent experimental conditions to ensure validity.

#### Quantification of MDA, SOD, TT, and CAT

MDA levels, as an indicator of lipid peroxidation, were measured using the thiobarbituric acid reactive substances (TBARS) method. Briefly, 0.5 ml of homogenized tissue supernatant was mixed with 2.5 ml of 20% trichloroacetic acid (TCA) and 1 ml of 0.67% thiobarbituric acid (TBA; Sigma-Aldrich). The mixture was heated at 95°C for 30 min in a water bath, then cooled to room temperature, and centrifuged at 3,000 rpm for 10 min. The absorbance of the resulting supernatant was measured at 532 nm. The concentration of MDA was calculated and expressed as nmol/mg tissue (Ohkawa et al., 1979; Akbari et al., 2023).

Total thiol groups were measured using Ellman's reagent [5,5′-dithiobis-(2-nitrobenzoic acid), DTNB; Sigma-Aldrich]. In brief, 50 µl of tissue homogenate was mixed with 1 ml of Tris-EDTA buffer, pH 8.6, and 50 µl of 10 mM DTNB. The mixture was incubated at room temperature for 15 min, and the absorbance was recorded at 412 nm. The concentration of thiol groups was calculated using a molar extinction coefficient of 13,600 M^−1^cm^−1^ and expressed as µmol/mg protein (Ellman, 1959).

SOD activity was quantified using a commercial colorimetric assay kit (e.g., Cayman Chemical, catalog #706002), following the manufacturer's protocol. This assay is based on the enzymatic production of superoxide radicals, which react with a tetrazolium salt (WST-1) to form a colored formazan dye measurable at 450 nm. SOD in the sample inhibits the formation of this dye by dismutating superoxide radicals. One unit of SOD activity is defined as the amount of enzyme that causes a 50% inhibition of the color development. Absorbance was measured and SOD activity was calculated using the provided standard curve.

CAT activity was determined spectrophotometrically by measuring the decomposition of hydrogen peroxide (H_2_O_2_) according to the method of Aebi (1984). Briefly, 50 µl of tissue homogenate was added to 2.95 ml of 30 mM H_2_O_2_ (Sigma-Aldrich) prepared in 50 mM phosphate buffer, pH 7.0. The decrease in absorbance was recorded at 240 nm over a period of 1 min. CAT activity was expressed as µmol H_2_O_2_ decomposed per minute per mg protein (Aebi, 1984; Akbari et al., 2023). All absorbance measurements were performed using a UV–Vis spectrophotometer (Model: UV-1600, Shimadzu).

### Statistical analyses

The data were analyzed using GraphPad Prism software (version 8.4.3). Statistical evaluation was carried out through one-way ANOVA, followed by Tukey's post hoc test for multiple comparisons. The results are presented as the mean ± standard error of the mean, with statistical significance defined at *α* = 0.05, representing a 95% confidence interval.

## Results

### Thiamine mitigates anxiety-like behaviors associated with nicotine withdrawal

**Figure 2.**
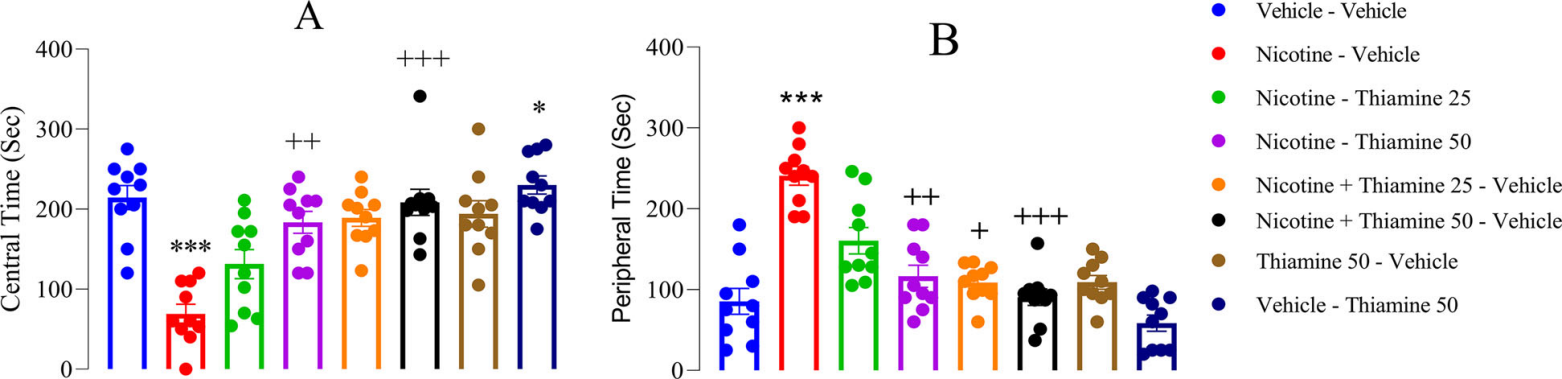
Effects of thiamine on anxiety-related behaviors in the open field test (OFT). ***A***, Time spent in the central area of the arena; (***B***) time spent in the peripheral area. Data are expressed as mean ± SEM (*n* = 10). Significant differences: *p < 0.05 and ***p < 0.001 compared with the vehicle–vehicle group; ^+^*p* < 0.05, ^++^*p* < 0.01, and ^+++^*p* < 0.001 compared with the nicotine–vehicle group.

The OFT revealed that nicotine withdrawal induced anxiety-like behaviors, as indicated by a significant decrease in time spent in the central area of the arena (*F*_(7,54)_ = 44.59, *p* < 0.001; Fig. 2*A*) and a corresponding increase in exploratory activity within the peripheral zones (*F*_(7,54)_ = 21.23, *p* < 0.001; Fig. 2*B*). Thiamine administration at 50 mg/kg during both nicotine exposure and withdrawal significantly increased central area exploration compared with the nicotine-vehicle group (*p* < 0.001; Fig. 2*A*). Additionally, thiamine at 50 mg/kg alone during P42–63 significantly increased central time compared with the vehicle-vehicle group (*p* < 0.05; Fig. 2*A*). Similarly, thiamine at 25 mg/kg during nicotine withdrawal and at 50 mg/kg during both exposure and withdrawal significantly reduced peripheral exploration compared with the nicotine-vehicle group (*p* < 0.05, *p* < 0.01, and *p* < 0.001, respectively; Fig. 2*B*).

**Figure 3.**
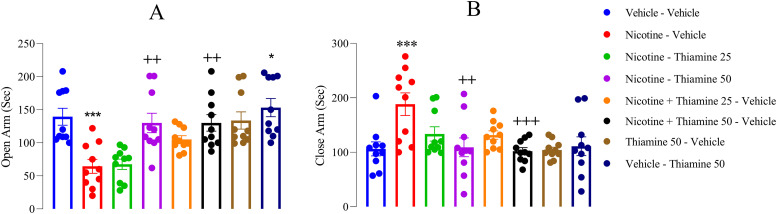
Effects of thiamine on anxiety-related behaviors in the elevated plus maze (EPM). ***A***, Time spent in the open arms; (***B***) time spent in the closed arms. Data are shown as mean ± SEM (*n* = 10). Significant differences: *p < 0.05 and ***p < 0.001 compared with the vehicle–vehicle group; ^+^*p* < 0.05, ^++^*p* < 0.01, and ^+++^*p* < 0.001 compared with the nicotine–vehicle group.

Findings from the EPM supported the OFT results, with nicotine withdrawal significantly decreasing time in the open arms (*F*_(7,54)_ = 35.72, *p* < 0.001; Fig. 3*A*) and increasing time in the closed arms (*F*_(7,54)_ = 31.68, *p* < 0.001; Fig. 3*B*) compared with the vehicle-vehicle group. Notably, thiamine at 50 mg/kg during both nicotine exposure and withdrawal significantly increased time spent in the open arms (*p* < 0.01; Fig. 3*A*) and reduced time in the closed arms (*p* < 0.01 and *p* < 0.001, respectively; Fig. 3*B*) relative to the nicotine-vehicle group. However, thiamine at 50 mg/kg alone did not significantly alter open or closed arm time compared with the vehicle-vehicle group.

### Thiamine mitigates depression-like behaviors associated with nicotine withdrawal

**Figure 4.**
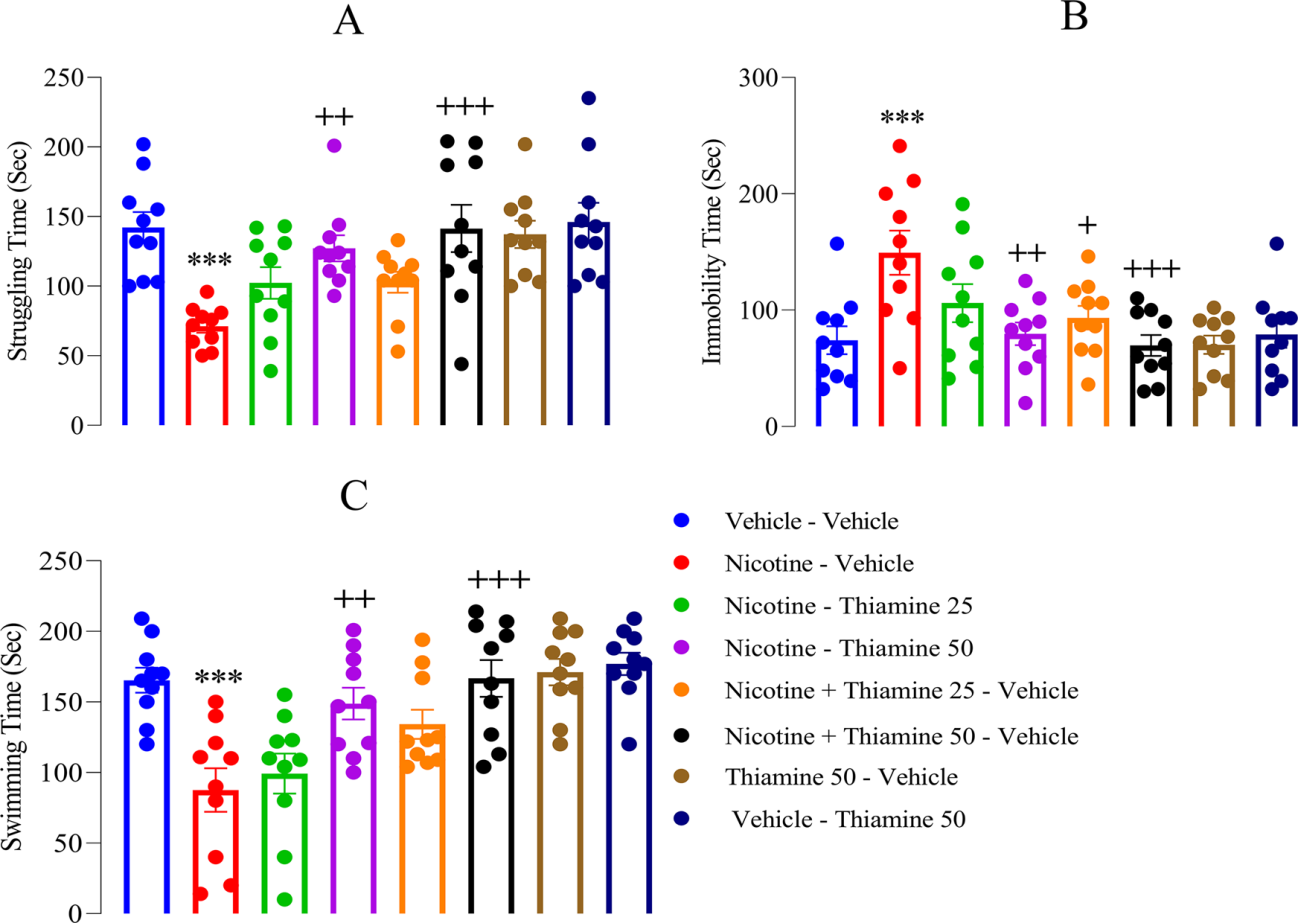
Effects of thiamine on depressive-like behaviors in the forced swim test. ***A***, Time spent struggling; (***B***) time spent immobile; (***C***) time spent swimming. Data are represented as mean ± SEM (*n* = 10). Significant differences: ***p < 0.001 compared with the vehicle–vehicle group; ^+^*p* < 0.05, ^++^*p* < 0.01, and ^+++^*p* < 0.001 compared with the nicotine–vehicle group.

In the FST, nicotine withdrawal significantly increased depressive-like behaviors, as evidenced by a reduction in struggling time (*F*_(7,54)_ = 41.16, *p* < 0.001; Fig. 4*A*), an increase in immobility (*F*_(7,54)_ = 35.42, *p* < 0.001; Fig. 4*B*), and a decrease in swimming time (*F*_(7,54)_ = 47.32, *p* < 0.001; Fig. 4*C*) compared with the vehicle-vehicle group. Thiamine administration at 25 mg/kg during nicotine withdrawal significantly reduced immobility time (*p* < 0.01; Fig. 4*B*). Additionally, thiamine at 50 mg/kg, both during nicotine exposure and withdrawal, significantly increased struggling (*p* < 0.01 and *p* < 0.001, respectively; Fig. 4*A*) and swimming times (*p* < 0.01 and *p* < 0.001, respectively; Fig. 4*C*), while reducing immobility (*p* < 0.01 and *p* < 0.001, respectively; Fig. 4*B*) compared with the nicotine-vehicle group. However, thiamine at 50 mg/kg alone did not produce significant behavioral changes in the FST.

### Thiamine mitigates anhedonia-like behaviors associated with nicotine withdrawal

**Figure 5.**
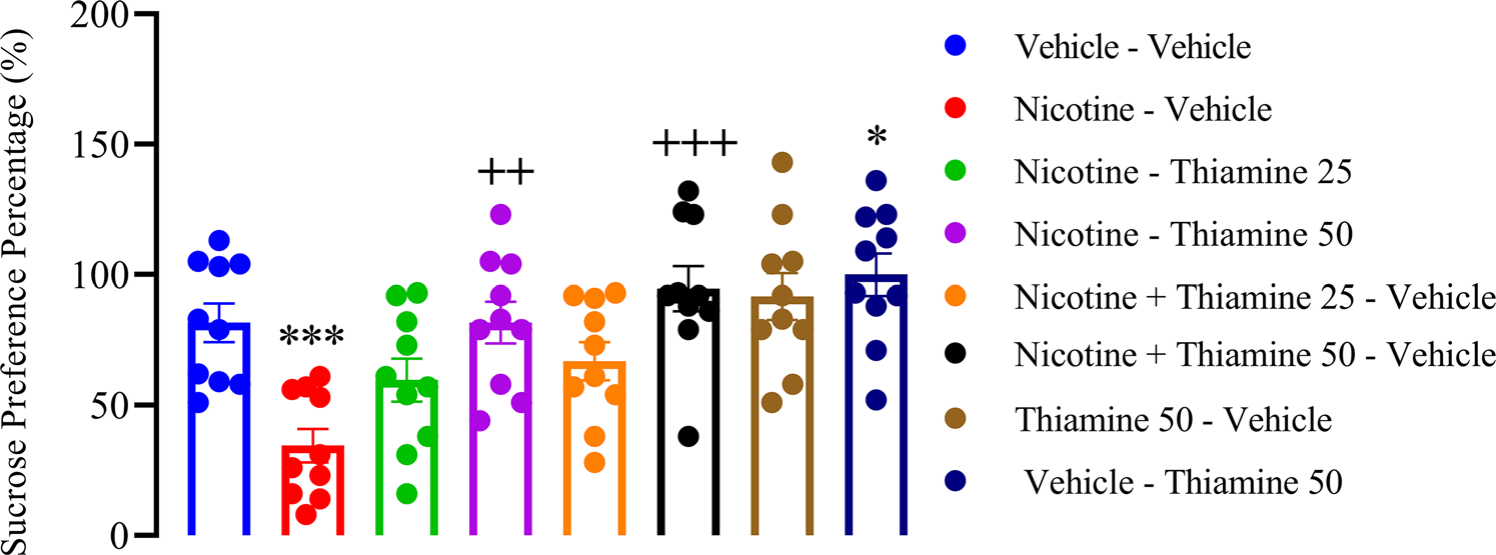
Effects of thiamine on anhedonia-like behaviors in the sucrose preference test (SPT). Data are shown as mean ± SEM (*n* = 10). Significant differences: *p < 0.05 and ***p < 0.001 compared with the vehicle–vehicle group; ^+^*p* < 0.01 and ^++^*p* < 0.001 compared with the nicotine–vehicle group.

The SPT results indicated that nicotine withdrawal induced anhedonia-like behaviors, as reflected by a significant decrease in sucrose preference compared with the vehicle-vehicle group (*F*_(7,54)_ = 40.36, *p* < 0.001; Fig. 5). Notably, thiamine treatment at 50 mg/kg, administered both during nicotine exposure and throughout withdrawal, significantly increased sucrose preference relative to the nicotine-vehicle group (*p* < 0.01 and *p* < 0.001, respectively; Fig. 5). Additionally, thiamine administration alone at 50 mg/kg during P42–63 significantly enhanced sucrose preference compared with the vehicle-vehicle group (*p* < 0.05; Fig. 5).

### Thiamine modulates oxidative stress parameters associated with nicotine withdrawal

**Figure 6.**
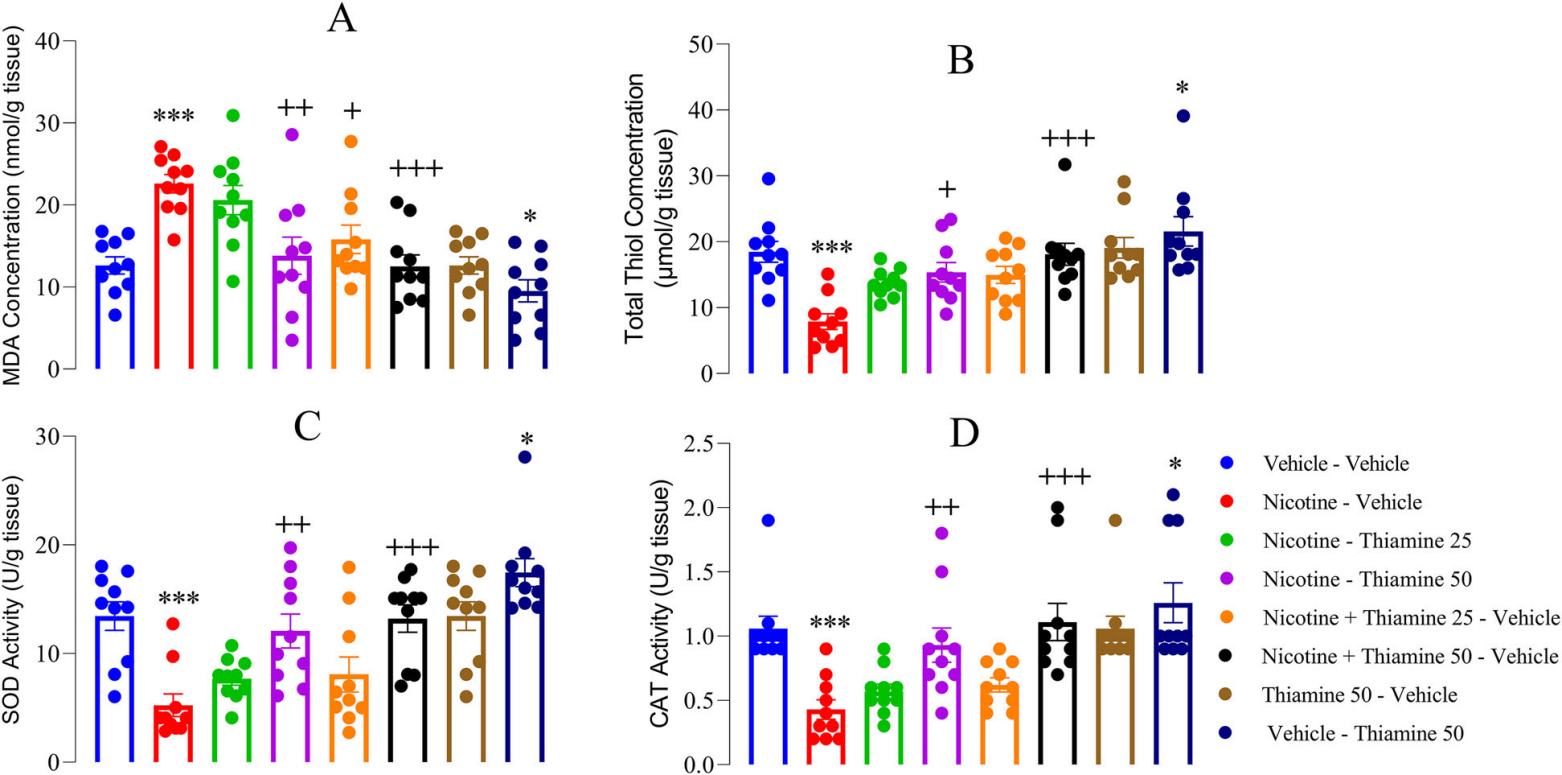
Effects of thiamine on oxidative stress markers in the prefrontal cortex. ***A***, Malondialdehyde (MDA) levels; (***B***) total thiol (TT) levels; (***C***) superoxide dismutase (SOD) activity; (***D***) catalase (CAT) activity. Data are presented as mean ± SEM (*n* = 10). Significant differences: *p < 0.05 and ***p < 0.001 compared with the vehicle–vehicle group; ^+^*p* < 0.05, ^++^*p* < 0.01, and ^+++^*p* < 0.001 compared with the nicotine–vehicle group.

Biochemical analyses revealed that nicotine withdrawal significantly disrupted oxidative stress homeostasis in the PFC. This disruption was characterized by a marked increase in MDA levels (*F*_(7,54)_ = 46.25, *p* < 0.001; Fig. 6*A*), alongside decreased thiol concentrations (*F*_(7,54)_ = 33.15, *p* < 0.001; Fig. 6*B*) and reduced enzymatic activities of SOD (*F*_(7,54)_ = 28.75, *p* < 0.001; Fig. 6*C*) and CAT (*F*_(7,54)_ = 33.27, *p* < 0.001; Fig. 6*D*) compared with the vehicle-vehicle group. Notably, coadministration of thiamine at 25 mg/kg significantly decreased MDA levels (*p* < 0.05; Fig. 6*A*). Furthermore, thiamine treatment at 50 mg/kg during both nicotine exposure and withdrawal led to significant reductions in MDA levels (*p* < 0.05 and *p* < 0.001; Fig. 6*A*) and increases in thiol concentrations (*p* < 0.05 and *p* < 0.005; Fig. 6*B*) and SOD activity (*p* < 0.01 and *p* < 0.001; Fig. 6*C*). Similarly, CAT activity was significantly elevated compared with the nicotine-vehicle group (*p* < 0.01 and *p* < 0.001; Fig. 6*D*). Additionally, thiamine administration at 50 mg/kg alone from P42–63 significantly reduced MDA levels while enhancing thiol concentrations and SOD and CAT activities compared with the vehicle-vehicle group (*p* < 0.05; Fig. 6*A–D*).

### Thiamine ameliorates neurochemical alterations associated with nicotine withdrawal

**Figure 7.**
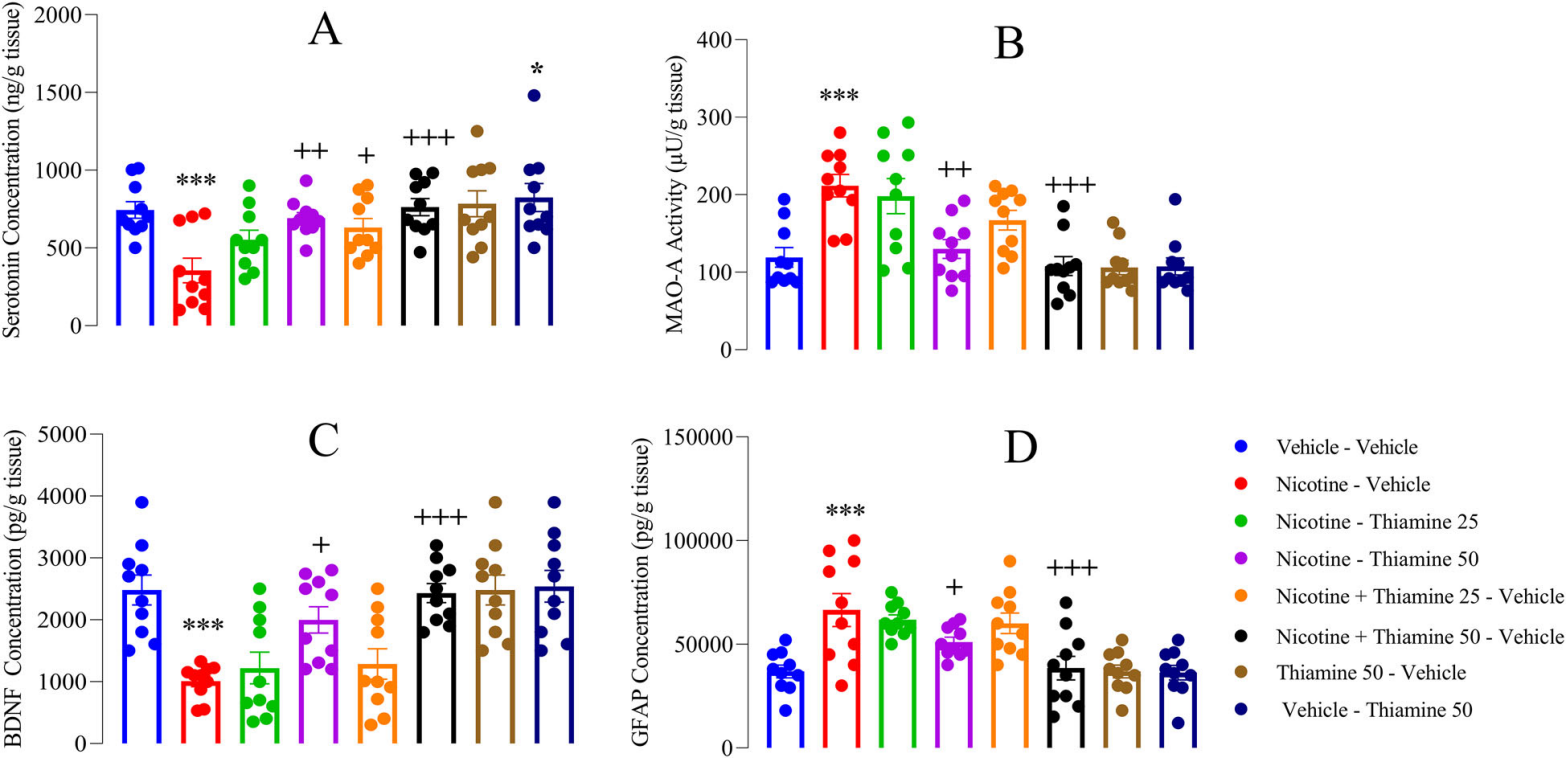
Effects of thiamine on cortical levels of neurotransmitters and biomarkers. ***A***, Serotonin levels; (***B***) monoamine oxidase-A (MAO-A) activity; (***C***) brain-derived neurotrophic factor (BDNF) levels; (***D***) glial fibrillary acidic protein (GFAP). Data are expressed as mean ± SEM (*n* = 10). Significant differences: *p < 0.05 and ***p < 0.001 compared with the vehicle–vehicle group; ^+^*p* < 0.05, ^++^*p* < 0.01, and ^+++^*p* < 0.001 compared with the nicotine–vehicle group.

Nicotine withdrawal was associated with a significant decrease in serotonin levels (*F*_(7,54)_ = 99.65, *p* < 0.001; Fig. 7*A*), along with a pronounced increase in MAO-A activity (*F*_(7,54)_ = 129.65, *p* < 0.001; Fig. 7*B*). Additionally, there was a marked reduction in BDNF levels (*F*_(7,54)_ = 43.95, *p* < 0.001; Fig. 7*C*) and an increase in GFAP expression (*F*_(7,54)_ = 10.65, *p* < 0.001; Fig. 7*D*) compared with the vehicle-vehicle group. Administration of thiamine at 25 mg/kg during nicotine exposure significantly increased serotonin levels (*p* < 0.05; Fig. 7*A*). Furthermore, thiamine treatment at 50 mg/kg during both nicotine exposure and withdrawal effectively elevated serotonin levels (*p* < 0.01 and *p* < 0.001, respectively; Fig. 7*A*), decreased MAO-A activity (*p* < 0.01 and *p* < 0.001, respectively; Fig. 7*B*), restored BDNF levels (*p* < 0.05 and *p* < 0.001, respectively; Fig. 7*C*), and reduced GFAP expression compared with the nicotine-vehicle group (*p* < 0.05 and *p* < 0.001, respectively; Fig. 7*D*). Notably, thiamine administration at 50 mg/kg alone from P42 to 63 resulted in a significant increase in serotonin levels compared with the vehicle-vehicle group (*p* < 0.05; Fig. 7*A*).

## Discussion

Although nicotine dependence and withdrawal are known to be associated with behavioral and neurochemical disturbances in both humans and animals (Haidary et al., 2024; Hossaini et al., 2025a), nicotine continues to be widely consumed by adolescents (Beckmann et al., 2024). Importantly, low doses of nicotine are insufficient to elicit spontaneous withdrawal symptoms such as anxiety-like behavior. These observations suggest that the extent of nicotine dependence in rats is closely linked to both the administered dose and the duration of exposure (Chellian et al., 2021). High-dose nicotine exposure during adolescence leads to significant and enduring changes in behavioral responses to addictive substances in adulthood (Slotkin et al., 2014; Renda et al., 2020). This exposure is associated with neurobehavioral alterations including heightened anxiety-like behaviors and disruptions in reward processing (Slawecki et al., 2003). Moreover, adolescent nicotine administration results in pronounced neurochemical changes, including reduced serotonin levels (Slotkin et al., 2014), impaired serotonin receptor function, and dysregulated cell signaling mediated by adenylyl cyclase (Xu et al., 2002). Structural brain changes have also been reported, including reductions in total gray matter (GM) volume, region-specific GM loss in the medial prefrontal cortex (mPFC), and altered GM volume covariation between the mPFC and other brain regions (Chen et al., 2025). Studies highlight that the developing brain is particularly susceptible to the neurotoxic effects of nicotine, resulting in long-lasting alterations to neurotransmitter systems that can increase the risk of emotional dysregulation and behavioral disorders later in life (Slotkin et al., 2014).

In this study, we focused on nicotine dependence rather than addiction, as the model was designed to evaluate behavioral and neurochemical changes related to withdrawal, such as anxiety, depression, and anhedonia. To effectively model nicotine dependence and subsequent withdrawal in adolescent rats, we administered subcutaneous nicotine at a dose of 2 mg/kg. Although this dose may be considered relatively high for rodent models, multiple studies have demonstrated that such dosing is necessary to reliably induce robust and measurable nicotine withdrawal symptoms (Amiry et al., 2023; Beheshti et al., 2025; Hossaini et al., 2025b). Importantly, rodent models typically require higher nicotine doses than humans due to interspecies differences in nicotine metabolism, receptor sensitivity, and pharmacokinetics. For instance, the plasma half-life of nicotine in rats is significantly shorter (∼1 h) than that in humans (2 h) (Chellian et al., 2021), necessitating higher dosing to achieve comparable receptor occupancy and plasma nicotine concentrations. Moreover, lower nicotine doses in adolescent and adult rats often fail to produce consistent spontaneous withdrawal symptoms, including anxiety-like behavior, hyperalgesia, and cognitive deficits (Chellian et al., 2021). With regard to translational relevance, it is important to recognize that nicotine withdrawal in humans occurs within a complex biopsychosocial context (Bruijnzeel, 2017; Chellian et al., 2022). Continued tobacco or e-cigarette use is not driven by withdrawal symptoms alone but also shaped by a dynamic interplay of biological factors (e.g., neuroadaptations; Bruijnzeel, 2012), psychological influences (e.g., stress, mood disorders, and habit formation; Pergadia et al., 2014; Bruijnzeel, 2017), and interconnectedness of factors (Torres et al., 2013; Isuru and Rajasuriya, 2019).

The findings demonstrate that nicotine withdrawal induces anxiety-like behaviors, as observed in the OFT and EPM. Specifically, nicotine withdrawal significantly increased anxiety-like behaviors, as observed in both the OFT and EPM. In the OFT, the significant reduction in time spent in the central area by the nicotine-vehicle group corroborates the established understanding that withdrawal from nicotine can lead to heightened anxiety levels in rats. This behavior aligns with previous research indicating that anxiety is often manifested through increased avoidance of open, exposed areas (Prut and Belzung, 2003; Sudakov et al., 2013; Hossaini et al., 2025a), reflecting the animals’ instinctive responses to perceived threats in their environment. Similarly, the EPM results showed decreased open-arm exploration and increased time in the closed arms, reinforcing the anxiogenic effects of nicotine cessation. These findings align with previous studies reporting that nicotine withdrawal disrupts anxiolytic behavioral patterns (Amiry et al., 2023; Rezaei Moghadam et al., 2023), likely due to alterations in neurotransmitter systems, including serotonergic signaling (Haidary et al., 2024; Hossaini et al., 2025a).

A key finding of this study is the anxiolytic effect of thiamine administration. Thiamine treatment significantly mitigated nicotine withdrawal-induced anxiety, as evidenced by an increase in central area exploration in the OFT and enhanced open arm time in the EPM. The effect was dose dependent, with 50 mg/kg producing the most robust behavioral improvements, in line with previous findings that thiamine influences serotonergic pathways (Mrowicka et al., 2023), which are critical for anxiety regulation (Albert et al., 2014). The anxiolytic effects of thiamine are consistent with existing literature that highlights the essential role of B vitamins, particularly thiamine, in brain health and mood regulation (Jahan-Mihan et al., 2024; Melki et al., 2024). Moreover, thiamine deficiency has been linked to various neurological disorders, emphasizing its importance in maintaining mental well-being (Fattal-Valevski, 2011). Research also indicates that thiamine supplementation may improve cognitive function in patients with neurodegenerative diseases, suggesting a strong connection between thiamine levels and mental health (Iskra and Trufanov, 2023). These findings collectively support the potential of thiamine as a therapeutic agent for managing anxiety related to nicotine withdrawal.

Interestingly, thiamine alone at 50 mg/kg (without nicotine exposure) also increased center time in the OFT, suggesting potential intrinsic anxiolytic properties, indicating that thiamine itself has intrinsic anxiolytic effects independent of nicotine exposure or withdrawal. Additionally, studies have found that better thiamine levels are linked to improved clarity, composure, and energy, highlighting its importance in cognitive and emotional processes (Benton et al., 1997), supporting the idea that thiamine may have intrinsic effects on anxiety. Yet, more in-depth investigations are needed to uncover the exact mechanisms at play.

This study also found that nicotine withdrawal induces depression-like behaviors, as demonstrated in the FST. Specifically, nicotine withdrawal resulted in a significant decrease in struggling time, an increase in immobility, and a reduction in swimming time, all of which are hallmark indicators of behavioral despair commonly observed in depressive-like states (Yankelevitch-Yahav et al., 2015). Thiamine administration mitigated nicotine withdrawal-induced depression-like behaviors. Thiamine at 25 mg/kg during nicotine withdrawal significantly reduced immobility time, indicating an antidepressant-like effect. More notably, 50 mg/kg of thiamine, administered both concurrently with nicotine and throughout withdrawal, significantly increased struggling and swimming behaviors while reducing immobility, suggesting a stronger therapeutic effect at higher doses. These findings suggest that thiamine may counteract the behavioral despair induced by nicotine withdrawal, potentially through its neuroprotective and neuromodulatory properties. The results indicated that nicotine withdrawal induces anhedonia-like behaviors, as evidenced by a significant decrease in sucrose preference in the SPT. This finding is consistent with existing literature that underscores the impact of substance withdrawal on mood and reward processing, highlighting how nicotine cessation can lead to negative emotional states (Alkhlaif et al., 2017; Murdaugh et al., 2024). Importantly, thiamine administration at 50 mg/kg, significantly attenuated anhedonia-like behaviors, as reflected by an increase in sucrose preference. This effect was observed both when thiamine was administered concurrently with nicotine and throughout the withdrawal period, suggesting a protective role against withdrawal-induced reward deficits. Moreover, thiamine at 50 mg/kg alone (without nicotine exposure) also significantly enhanced sucrose preference, indicating that its effects may extend beyond nicotine withdrawal to more generalized reward-enhancing properties. These results support the hypothesis that thiamine is essential for emotional regulation and mood stabilization (Mrowicka et al., 2023). Nonetheless, additional research is essential to elucidate the precise mechanisms underlying these effects.

The PFC plays a pivotal role in the regulation of anxiety and depression by modulating emotional responses and executive functions (Fitzgerald et al., 2019; Kenwood et al., 2022). Dysregulation of PFC activity has been consistently associated with the pathophysiology of anxiety disorders and major depressive disorder (Shiba et al., 2016).

Notably, nicotine exposure has been shown to induce considerable oxidative stress in the PFC, as evidenced by increased MDA levels, decreased thiol concentrations, and reduced enzymatic activities of SOD and CAT. These findings align with previous studies indicating that nicotine withdrawal triggers excessive oxidative stress, leading to neuronal damage, neuroinflammation, and impaired cognitive and affective functions (Haidary et al., 2024; Hossaini et al., 2025a). Given the critical role of oxidative stress in neuropsychiatric disorders, such as anxiety, depression, and cognitive impairments, the observed biochemical alterations further support the notion that oxidative damage contributes to the behavioral disturbances associated with nicotine withdrawal (Fedoce et al., 2018; Dash et al., 2024). Importantly, thiamine administration mitigated nicotine withdrawal-induced oxidative stress. Thiamine at 25 mg/kg significantly reduced MDA levels and increased thiol concentrations, suggesting an initial protective effect against lipid peroxidation and oxidative imbalance. More notably, thiamine at 50 mg/kg, administered both during nicotine exposure and withdrawal, further enhanced antioxidant defenses by significantly decreasing MDA levels while simultaneously increasing thiol concentrations and restoring the activities of SOD and CAT. These findings indicate that higher doses of thiamine offer greater neuroprotective benefits, potentially by enhancing endogenous antioxidant defense mechanisms. The mechanisms underlying thiamine's protective effects against oxidative stress may involve its role in mitochondrial function, redox homeostasis, and neuroprotection. Thiamine is a critical cofactor for several enzymes involved in energy metabolism and oxidative phosphorylation, and its deficiency has been linked to increased oxidative stress and neurodegeneration (Mrowicka et al., 2023). By enhancing mitochondrial function and reducing oxidative damage, thiamine may help prevent neuronal injury and dysfunction associated with nicotine withdrawal. Additionally, thiamine's ability to increase SOD and CAT activity suggests that it strengthens endogenous antioxidant defenses, further reducing oxidative damage and promoting neuronal resilience. The antioxidant effects of thiamine align with existing literature that underscores its role as an antioxidant agent. Additionally, thiamine has been shown to reduce oxidative damage in various experimental settings (Lukienko et al., 2000), including diabetes and liver disease, where it alleviates oxidative stress by modulating metabolic pathways (Kohda et al., 2019). Interestingly, thiamine at 50 mg/kg alone (without nicotine exposure) also significantly improved oxidative stress markers compared with the vehicle group, indicating that its effects are not limited to withdrawal-induced oxidative damage but may also provide baseline neuroprotective benefits. This suggests that thiamine supplementation could serve as a potential strategy for maintaining oxidative balance and preventing neurodegenerative processes, even in the absence of external stressors.

The present study also reveals that nicotine withdrawal induces significant neurochemical alterations, including a reduction in serotonin levels, an increase in MAO-A activity, and decreased BDNF levels. These findings suggest that nicotine withdrawal is associated with serotonergic dysregulation and neurotrophic impairment which may contribute to the behavioral and affective disturbances commonly observed during withdrawal. These observed neurochemical alterations are consistent with the dysregulation of serotonergic pathways during nicotine withdrawal. This dysregulation is particularly concerning, as serotonin plays a critical role in mood regulation, and its depletion is frequently associated with anxiety and depressive-like symptoms (Amiry et al., 2023). The increase in MAO-A activity further exacerbates this effect, as MAO-A degrades serotonin, leading to further reductions in its availability and exacerbating withdrawal-related mood disturbances. These findings align with previous research suggesting that nicotine dependence modulates serotonergic neurotransmission, and withdrawal from chronic nicotine exposure results in serotonin depletion and heightened monoaminergic degradation, contributing to mood instability and anhedonia (Hitsman et al., 2007). Importantly, thiamine administration was found to mitigate these neurochemical disruptions. Thiamine at 25 mg/kg significantly increased serotonin levels, suggesting a partial restoration of serotonergic function. More notably, thiamine at 50 mg/kg, administered both during nicotine exposure and withdrawal, effectively elevated serotonin levels, reduced MAO-A activity, restored BDNF levels, and decreased GFAP expression. These findings highlight the neuroprotective and neuromodulatory properties of thiamine, particularly in the context of nicotine withdrawal-induced neurochemical deficits. The ability of thiamine to increase serotonin levels while simultaneously reducing MAO-A activity suggests a potential regulatory effect on monoaminergic neurotransmission. Thiamine has been implicated in neurotransmitter synthesis and mitochondrial function, both of which play crucial roles in maintaining serotonergic homeostasis (Mrowicka et al., 2023). By reducing MAO-A activity, thiamine may help preserve serotonin availability, counteracting the serotonergic depletion associated with nicotine withdrawal. Additionally, the restoration of BDNF levels suggests that thiamine supports neuroplasticity and neuronal survival, as BDNF is essential for synaptic plasticity, mood regulation, and cognitive function (Lu et al., 2014; Begni et al., 2016).

Our study also demonstrated significant neuroinflammation during nicotine withdrawal, as evidenced by elevated GFAP expression in the PFC, which may contribute to the disruption of serotonergic signaling, a key neurotransmitter system critically involved in mood regulation (Hossaini et al., 2025b). GFAP is an intermediate filament protein that is released by activated astrocytes within the central nervous system and is widely recognized as a significant biomarker for neuroinflammation and astrocyte activation (Leipp et al., 2024). Research on the association between neuron–astrocyte interactions in depression indicates that astrogliosis, characterized by elevated GFAP levels, is correlated with disrupted monoamine signaling. This specifically implicates serotonin dysfunction as a key factor in mood disturbances (Zhou et al., 2019; Yao et al., 2023). Importantly, thiamine administration led to a reduction in GFAP expression within the PFC, indicating attenuation of astrocyte activation and neuroinflammation. This finding further supports thiamine's neuroprotective properties, suggesting its potential role in preserving neuronal integrity and facilitating functional recovery.

Interestingly, thiamine at 50 mg/kg alone (without nicotine exposure) also led to a significant increase in serotonin levels, suggesting that its neuromodulatory effects extend beyond nicotine withdrawal. This finding implies that thiamine supplementation may enhance serotonergic function even under baseline conditions, potentially offering therapeutic benefits for individuals with mood disorders characterized by serotonergic dysfunction. This is supported by the fact that thiamine is essential for the synthesis of neurotransmitters, including serotonin, which significantly influences anxiety and mood. Research indicates that increased thiamine levels can enhance serotonergic activity, potentially leading to reduced anxiety-like behaviors (Mrowicka et al., 2023). However, more studies are required to clarify the specific mechanisms involved.

While the findings of this study are promising, some limitations should be acknowledged. Firstly, the use of twice daily subcutaneous nicotine injections to induce dependence, while effective in producing withdrawal-related neurobehavioral changes in rodents, does not fully replicate the pharmacokinetic profile of human nicotine consumption. As described by Benowitz (2010), habitual smokers typically experience ∼20 distinct nicotine peaks per day, resulting in rapid and dynamic fluctuations in plasma nicotine levels. In contrast, our dosing regimen generates a more sustained exposure pattern and lacks the high-frequency oscillations characteristic of real-world tobacco or e-cigarette use. Although this protocol is widely accepted in preclinical studies and provides a controlled framework for assessing withdrawal mechanisms (Pandey et al., 2001; Campos et al., 2008; Chellian et al., 2021), this pharmacokinetic discrepancy represents a translational limitation. Future studies should employ more physiologically relevant delivery systems, such as inhalation models or programmable infusions, which may better simulate human nicotine intake patterns and enhance the ecological validity of the findings.

The second limitation of the current study is the reliance solely on GFAP levels to assess neuroinflammation. While GFAP is a well-established marker of astrocyte activation (Agnello et al., 2025; Youn et al., 2025), neuroinflammation is a complex process involving multiple cell types and signaling pathways, including microglial activation (DiSabato et al., 2016) and pro-inflammatory cytokine release (Müller and Di Benedetto, 2025). Therefore, exclusive measurement of GFAP may provide an incomplete picture of the inflammatory response. Future studies should incorporate additional markers, such as Iba1 for microglia, as well as cytokines like IL-1β and TNF-α, which would offer a more comprehensive assessment of neuroinflammatory changes associated with nicotine withdrawal and related interventions (Kumar et al., 2024). Recognizing this limitation is important for contextualizing our findings and guiding more detailed mechanistic investigations.

Additionally, the absence of a positive control, such as varenicline, limits the ability to compare the efficacy of thiamine with established pharmacotherapies for nicotine dependence. Varenicline, a partial agonist of nicotinic acetylcholine receptors, has been shown to reduce withdrawal symptoms and cravings and could serve as a benchmark for evaluating new therapeutic agents. Future studies should incorporate positive control groups to provide a clearer context for assessing thiamine's efficacy relative to standard treatments. While thiamine appears effective in mitigating symptoms of nicotine withdrawal, its precise mechanism of action remains to be fully elucidated. Further investigation into how thiamine modulates serotonin pathways, oxidative stress responses, and neuroinflammatory markers would enhance our understanding of its therapeutic potential. Additionally, studies exploring different dosages, treatment durations, and combinations with other pharmacological agents could help optimize the clinical application of this treatment. Finally, clinical trials will be essential to determine whether thiamine supplementation is effective and safe for managing nicotine withdrawal in human populations.

### Conclusion

Thiamine appears to offer a promising therapeutic strategy for mitigating the behavioral and neurochemical alterations associated with nicotine withdrawal. Its effects on anxiety-like, depression-like, and anhedonia-like behaviors, along with its ability to modulate oxidative stress and restore neurochemical balance, highlight its potential as a treatment for nicotine dependence. However, the inclusion of a positive control and further mechanistic studies are necessary to fully evaluate thiamine's efficacy and its place within the broader landscape of treatments for nicotine withdrawal and dependence.

## Data Availability

The data generated during this study are available upon request from the corresponding author.

## Synthesis

Reviewing Editor: Katrina Choe, McMaster University Decisions are customarily a result of the Reviewing Editor and the peer reviewers coming together and discussing their recommendations until a consensus is reached. When revisions are invited, a fact-based synthesis statement explaining their decision and outlining what is needed to prepare a revision will be listed below. The following reviewer(s) agreed to reveal their identity: NONE.

This study examined the therapeutic effects of thiamine on nicotine withdrawal-induced anxiety, anhedonia and depression in rats. The authors report that nicotine withdrawal induced increased oxidative stress and neuroinflammatory effects alongside expected behavioral phenotypes in rats. In contrast, thiamine administration during both nicotine exposure and withdrawal periods alleviated behavioural and biochemical phenotypes. Both reviewers expressed that this study advanced the field by demonstrating potentially protective effects of thiamine on nicotine withdrawal-associated behavioural phenotypes, as well as other depression- and anxiety-like disorders. They also commented on some shortcomings with the manuscript, including typographical errors, choice of wording (e.g. addiction vs. dependence) and missing methodological information such as the timeline of experiments, justification for nicotine dose and routes of administration. Please see below for specific comments from the reviewers.

Reviewer 1

In this experiment the authors gave nicotine to rats for three weeks, allowed a 3-week withdrawal period, and gave animals 0, 25, or 50 mg/kg thiamine during either the nicotine or withdrawal phases. They found that thiamine ameliorated almost all the behavioral and biochemical measures they took. In all it seems to be a straightforward paper. I did not spot any major design flaws. However the manuscript seems to be incomplete, there are many typos throughout (including the figures) and several cases of missing information marked by "(???)". The timeline of experimental tests is also unclear. When did the tests occur relative to the end of nicotine administration? How long were the tests? What part of the light cycle did they occur? In general a more detailed explanation of the timeline is needed before I can be convinced that nicotine exposure still had an observable withdrawal syndrome after three weeks of cessation of treatment.

Statistics: The statistics are described very briefly. It appears that there was basically a 1-way ANOVA comparing all groups. The figures show 8 bars, but the degrees of freedom suggest only 6 groups were compared. I think it would make more sense to do a factorial ANOVA with nicotine/saline and thiamine dose as separate factors.

Reviewer 2

The authors demonstrated the effects of Thiamine on nicotine withdrawal effect in adolescent male rats. via modulation of serotonin, BDNF, oxidative stress, and neuroinflammation. Thiamine showed anti-depressive- and -anxiety-like behaviors in male rats. In addition, thiamine significantly attenuated serotonin levels and oxidative stress (MDA, SOD, and CAT levels) in the PFC of nicotine withdrawal rats. Therefore, thiamine has a therapeutic potential on depression-, anxiety-, or anhedonia-like disorder from nicotine withdrawal. However, there are some concerns related to experiments.

Major

1. What is the difference of nicotine addiction and nicotine dependence? Did the authors measure depression- or anxiety-like behaviors as the factors of physical dependence? If so, the authors consider the use of word: nicotine addiction or nicotine dependence in the manuscript.

2. Line 85-86, Is the dose of 2 mg/kg nicotine for adult nicotine intake? Dose of 2 mg/kg nicotine is strong in adolescent phase. Adolescent brain (PND 21~42) is still developing and 2 mg/kg nicotine is fatal dose in adolescent.

3. Please clarify the route of nicotine administration in the manuscript. Why the authors used two injection routes for nicotine injection: IP and SC??? Page 4, line 85 vs. line 91.

4. In Figure 1, why the authors did not inject saline (s.c.) as a vehicle against nicotine during PND 21-42?

5. How long the animals were recorded locomotor activity? Please provide the detailed procedure for behavioral tests in the method sections.

6. Please clarify all experiment procedure and provide the detail information for used chemical, antibody, or assay kit in the method section.

7. Please add 'Figure 1' behind thiamine treatment in line 99.

8. Please provide why the authors targeted 'prefrontal cortex' regarding oxidative stress or neuroinflammation in nicotine withdrawal rats.

9. Regarding neuroinflammation, the authors measured GFAP level for activation of astrocyte in the PFC. In neuroinflammatory response by drugs, microglia activation is also important. Why the authors did not measure the activated microglia levels in the PFC (ex: Iba-1 expression) and key cytokine levels (IL-1β, IL-6, TNF-α.........) of neuroinflammation? It is lack measured only GFPA levels to express the neuroinflammatory esponse.

10. Please provide n values in all Figure legends.

Minor

1. Line 8: Change 'SC' to 's.c.' like i.p..

2. Line 11-12, Please provide brain area for biochemical analyses.

3. Line 75 - 76, please provide 'humidity condition'.

4. What is '???' in line 72, 77, and 79.

5. Line 79, please provide approved IACUC animal study numbers.

6. Line 84: 'subcutaneous (s.c.)' like 'intraperitoneal (i.p.)' at the same line.

7. Line 85, does 'total injection volume of 1 mL' mean '1 mL/kg' injection?

8. Line 85-86,

9. Line 89, the animals were given twice nicotine injection in a day. Please provide time interval.

10. How the authors selected '5 min' for locomotor activity test in 2-3-1 open field test? Five minute is too short.

11. Line, 139, FST were measured after EPM test?

12. Line 140, please change 'rodents' to 'rats'. And, please provide how long FST recorded in the method section.

13. Line 181, please provide detailed information for quantification of serotonin and BDNF levels. Information of serotonin antibody or ELISA kit. Provided MyBioSource (MBS713292) information is for MAO-A.

14. Line 199, please provide detailed information for SOD assay kit.

15. Line 206, statistical analyses section: Please change the last sentence. In the figures, there is a significant difference vs. control groups as P < 0.01 or P < 0.001.

16. Please delete 'down direction' in X-axis of all Figures.

## Author Response

Dear Dr. Katrina Choe Thank you for the opportunity to revise our manuscript titled "Thiamine Mitigates Nicotine Withdrawal Effects in Adolescent Male Rats: Modulation of Serotonin Metabolism, BDNF, Oxidative Stress, and Neuroinflammation," We appreciate the your feedback and have made the necessary revisions to address your comments.

Thank you once again for your consideration. I look forward to your feedback on the revised manuscript.

Sincerely, Response to Reviewers:

We appreciate the constructive feedback provided by the reviewers. We are pleased to hear that they recognize the significance of our findings regarding thiamine's protective effects against nicotine withdrawal-induced anxiety, anhedonia, and depression. We acknowledge the identified shortcomings, including typographical errors and wording choices such as "addiction" versus "dependence." We will carefully revise the manuscript to address these issues. Additionally, we will provide clearer methodological details, including the experimental timeline and justification for the nicotine dosage and administration routes. Thank you for your valuable insights, which will enhance the clarity and quality of our study.

Reviewer #1 Comment 1: In this experiment the authors gave nicotine to rats for three weeks, allowed a 3-week withdrawal period, and gave animals 0, 25, or 50 mg/kg thiamine during either the nicotine or withdrawal phases. They found that thiamine ameliorated almost all the behavioral and biochemical measures they took. In all it seems to be a straightforward paper. I did not spot any major design flaws. However the manuscript seems to be incomplete, there are many typos throughout (including the figures) and several cases of missing information marked by "(???)". The timeline of experimental tests is also unclear. When did the tests occur relative to the end of nicotine administration? How long were the tests? What part of the light cycle did they occur? In general a more detailed explanation of the timeline is needed before I can be convinced that nicotine exposure still had an observable withdrawal syndrome after three weeks of cessation of treatment.

Response: Thank you for your thorough review and positive feedback regarding our experimental design. We appreciate your observations about the effectiveness of thiamine in ameliorating behavioral and biochemical measures. We acknowledge the manuscript's shortcomings, including typographical errors and instances of missing information. We addressed these issues and ensured that all figures are corrected.

Regarding the missing information (???), we would like to clarify that, following the journal's guidelines, we refrained from including any identifying text or addresses that could reveal the authors' identities during the review process. The name of the university and the code of ethics, which were previously removed, have now been included in the revised version.

Regarding the experimental timeline, we provided a detailed explanation of when the tests occurred relative to the end of nicotine administration, the duration of the tests, and their timing within the light cycle. This additional information will clarify the presence of withdrawal symptoms after the three-week cessation period. The responses are highlighted in the revised manuscript Thank you for your constructive suggestions, which will help strengthen our manuscript.

Comment #2: The statistics are described very briefly. It appears that there was basically a 1-way ANOVA comparing all groups. The figures show 8 bars, but the degrees of freedom suggest only 6 groups were compared. I think it would make more sense to do a factorial ANOVA with nicotine/saline and thiamine dose as separate factors.

Response: We appreciate your valuable feedback on the statistical analysis. To clarify, we conducted a one-way ANOVA followed by Tukey's post hoc test for multiple comparisons. Additionally, the discrepancy in the reported degrees of freedom was an inadvertent typographical error, which has now been corrected in the revised version. Your suggestions are greatly appreciated and will enhance the clarity and rigor of our statistical methods. The responses are highlighted in the revised manuscript Reviewer #2 Comment #1: What is the difference of nicotine addiction and nicotine dependence? Did the authors measure depression- or anxiety-like behaviors as the factors of physical dependence? If so, the authors consider the use of word: nicotine addiction or nicotine dependence in the manuscript.

Response: Thank you for your thoughtful questions regarding nicotine addiction and dependence. In our study, we focused specifically on nicotine dependence, which encompasses both physical and psychological aspects. We measured depression- and anxiety-like behaviors as indicators of physical dependence during the withdrawal phase.

We understand the importance of terminology and will ensure that our manuscript clearly distinguishes between nicotine addiction and dependence. We revised the text to reflect this distinction accurately while emphasizing our focus on nicotine dependence in the context of our findings. Your feedback is instrumental in enhancing the clarity of our work.

Comment 2#: Line 85-86, Is the dose of 2 mg/kg nicotine for adult nicotine intake? Dose of 2 mg/kg nicotine is strong in adolescent phase. Adolescent brain (PND 21~42) is still developing and 2 mg/kg nicotine is fatal dose in adolescent.

Response: Thank you for your important comments regarding the nicotine dosage used in our study. We acknowledge that a dose of 2 mg/kg may be considered high, particularly for adolescent rats, whose brains still develop during the postnatal days (PND 21-42). To clarify, the 2 mg/kg dosage was chosen based on prior studies that establish it as relevant for modeling adult nicotine intake. However, we recognize the potential implications for adolescent subjects and have revised the manuscript to address this concern. We provided additional context regarding this dosage's justification and relevance to our study's objectives. Your feedback is invaluable in ensuring the safety and accuracy of our experimental design (line 332 - 342).

Comment #3: Please clarify the route of nicotine administration in the manuscript. Why the authors used two injection routes for nicotine injection: IP and SC??? Page 4, line 85 vs. line 91.

Response: We appreciate your attention to the clarity of our manuscript. It should be noted that the previous wording was somewhat ambiguous. To clarify, nicotine was administered subcutaneously, while thiamine was administered intraperitoneally. This correction has been made in the current version of the manuscript. Thank you for your valuable feedback, which has helped improve our clarity (line 91).

Comment #4: In Figure 1, why the authors did not inject saline (s.c.) as a vehicle against nicotine during PND 21-42? Response: Thank you for your comment regarding Figure 1. We felt it was important to clarify that the study design included a saline vehicle for comparison; however, the timing was not presented clearly in the previous version. This has been corrected in the current manuscript to better illustrate the use of saline as a vehicle against nicotine during PND 21-42. Your feedback has been invaluable in enhancing the clarity of our presentation (figure 1).

Comment #5: How long the animals were recorded locomotor activity? Please provide the detailed procedure for behavioral tests in the method sections.

Response: Thank you for your suggestion. The animals' locomotor activity was recorded for a duration of 5 minutes following the administration of treatments. We provided a detailed description of the behavioral testing procedures in the methods section to ensure clarity and transparency. Your feedback is appreciated and will enhance the rigor of our methodology (Behavioral assessments).

Comment #6. Please clarify all experiment procedure and provide the detail information for used chemical, antibody, or assay kit in the method section.

Response: Thank you for your valuable feedback. We clarified the experimental procedures and provide detailed information regarding all chemicals, antibodies, and assay kits used in the methods section. This will ensure that our methodology is transparent and reproducible. Your suggestions are greatly appreciated and will strengthen our manuscript (Biochemical assessments).

Comment #7. Please add 'Figure 1' behind thiamine treatment in line 99.

Response: Thank you for your suggestion. We added a short sentence to refere to the figure 1 in the revised mauscript. Your attention to detail is appreciated (line 122).

Comment #8. Please provide why the authors targeted 'prefrontal cortex' regarding oxidative stress or neuroinflammation in nicotine withdrawal rats.

Response: Thank you for your insightful question. The rationale for targeting the prefrontal cortex in our study is included in the revised manuscript. We chose this region due to its critical role in cognitive function and its vulnerability to oxidative stress and neuroinflammation during nicotine withdrawal. Your feedback has helped us clarify this important aspect of our research (401-410).

Comment #9. Regarding neuroinflammation, the authors measured GFAP level for activation of astrocyte in the PFC. In neuroinflammatory response by drugs, microglia activation is also important. Why the authors did not measure the activated microglia levels in the PFC (ex: Iba-1 expression) and key cytokine levels (IL-1β, IL-6, TNF-α.........) of neuroinflammation? It is lack measured only GFPA levels to express the neuroinflammatory esponse.

Response: Thank you for your valuable feedback. We acknowledge that measuring additional indicators would provide a more comprehensive understanding of neuroinflammation. However, due to budget constraints and in alignment with our previous studies, we focused on GFAP levels as an indicator of astrocyte activation. We appreciate your suggestions and will consider them for future research to enhance our investigation of neuroinflammatory responses (line 401 - 410).

Comment # 10. Please provide n values in all Figure legends.

Response: Thank you for your suggestion. We included the n values in all figure legends to improve clarity and transparency in our data presentation. Your feedback is appreciatedu (figures).

Minor Comment #1: Line 8: Change 'SC' to 's.c.' like i.p.

Response: Thank you for your attention to detail. We changed 'SC' to 's.c.' to maintain consistency with the abbreviation for i.p. Your suggestion is appreciated (line 7).

Comment #2: Line 11-12, Please provide brain area for biochemical analyses.

Response: Thank you for your attention to detail. We provided brain area for biochemical analyses. Your suggestion is appreciated (line 11).

Comment #3: Line 75 - 76, please provide 'humidity condition'.

Response: Thank you for your request. We included the specific humidity conditions in the revised manuscript to provide a clearer context for our experimental setup. Your feedback is valuable (line 81).

Comment #4: What is '???' in line 72, 77, and 79.

Response: Thank you for your inquiry. The '???' referred to the name of the rats food company, the university, and the code of ethics. These details were removed in accordance with the journal's guidelines but have been included in the current version of the manuscript. Your feedback helped us clarify this aspect.

Comment #5: Line 79, please provide approved IACUC animal study numbers.

Response: Thank you for your suggestion. We included the approved IACUC animal study numbers to ensure compliance and transparency. Your input is greatly appreciated (line 85).

Comment #6: Line 84: 'subcutaneous (s.c.)' like 'intraperitoneal (i.p.)' at the same line.

Response: Thank you for your feedback. We meant to indicate that nicotin was injected subcutaneously and Thiamine intraperitoneally. We acknowledge that the text was unclear, and we have made corrections in the revised manuscript to clarify this, aligning the terminology for both injections. Your suggestion has been very helpful (line 91).

Comment #7: Line 85, does 'total injection volume of 1 mL' mean '1 mL/kg' injection? Response: Thank you for your question. Our goal for the final injection volume is one milliliter, which means that after calculating the doses, the drugs were adjusted to a total volume of one milliliter before injection. We appreciate your clarification and have adjusted the manuscript to ensure this is communicated (line 90).

Comment #8: Line 89, the animals were given twice nicotine injection in a day. Please provide time interval.

Response: Thank you for your feedback. We aded the time interval. Your suggestion has been very helpful (line 91 - 92).

Comment #9: How the authors selected '5 min' for locomotor activity test in 2-3-1 open field test? Five minute is too short.

Response: Thank you for your observation regarding the duration of the locomotor activity test. We selected a 5-minute interval based on previous studies that demonstrated this duration is sufficient to assess initial locomotor activity in the open field test. However, we acknowledge your concern and discuss the potential for longer observation times in the revised manuscript. Your input is greatly appreciated (line 146 - 155).

Comment #10: Line, 139, FST were measured after EPM test? Response: Thank you for your question. The Forced Swim Test was indeed conducted after the Elevated Plus Maze test. We clarified this sequence in the manuscript to avoid any confusion. Your feedback is valuable, and we appreciate your attention to detail.

Comment #11: Line 140, please change 'rodents' to 'rats'. And, please provide how long FST recorded in the method section.

Response: Thank you for your feedback. We did accordingly (line 188).

Comment #12: Line 181, please provide detailed information for quantification of serotonin and BDNF levels. Information of serotonin antibody or ELISA kit. Provided MyBioSource (MBS713292) information is for MAO-A (line 129-137).

Response: Thank you for your feedback. We did accordingly.

Comment #13: Line 199, please provide detailed information for SOD assay kit.

Response: Thank you for your comment. We would like to clarify that we did not use a commercial kit to measure Superoxide Dismutase. Instead, we employed a colorimetric method based on our previous studies and those of other authors. We will provide a more detailed explanation of this method in the revised manuscript. Your suggestion is greatly appreciated.

Comment #14: Line 206, statistical analyses section: Please change the last sentence. In the figures, there is a significant difference vs. control groups as P < 0.01 or P < 0.001.

Response: Thank you for your feedback. We did accordingly.

Comment #15: Please delete 'down direction' in X-axis of all Figures (figures).

Response: Thank you for your feedback. We did accordingly.
